# Psychosocial stress, interpersonal sensitivity, and social withdrawal in clinical high risk for psychosis: a systematic review

**DOI:** 10.1038/s41537-023-00362-z

**Published:** 2023-06-17

**Authors:** A. Georgiades, A. Almuqrin, P. Rubinic, K. Mouhitzadeh, S. Tognin, A. Mechelli

**Affiliations:** 1 Department of Psychosis Studies, Institute of Psychiatry, Psychology, and Neuroscience (IoPPN), King’s College London, London, UK; 2 Brent Early Intervention Service, CNWL, NHS Foundation Trust, 27-29 Fairlight Avenue, London, NW10 8AL UK

**Keywords:** Psychosis, Human behaviour

## Abstract

Stress has repeatedly been implicated in the onset and exacerbation of positive symptoms of psychosis. Increasing interest is growing for the role of psychosocial stress in the development of psychosis symptoms in individuals at Clinical High Risk (CHR) for psychosis. A systematic review was therefore conducted to summarize the existing evidence base regarding psychosocial stress, interpersonal sensitivity, and social withdrawal in individuals at CHR for psychosis. An electronic search of Ovid (PsychINFO, EMBASE, MEDLINE, and GLOBAL HEALTH) was conducted until February 2022. Studies that examined psychosocial stress in CHR were included. Twenty-nine studies were eligible for inclusion. Psychosocial stress, interpersonal sensitivity, and social withdrawal were higher in CHR individuals compared to healthy controls and there was some evidence of their association with positive symptoms of psychosis. Two types of psychosocial stressors were found to occur more frequently with CHR status, namely daily stressors, and early and recent trauma, while significant life events did not appear to be significant. Greater exposure to psychosocial stress, emotional abuse, and perceived discrimination significantly increased risk of transition to psychosis in CHR. No studies examined the role of interpersonal sensitivity on transition to psychosis in CHR. This systematic review provides evidence for the association of trauma, daily stressors, social withdrawal, and interpersonal sensitivity with CHR status. Further studies investigating the impact of psychosocial stress on psychosis symptom expression in individuals at CHR and its effects on transition to psychosis are therefore warranted.

## Introduction

Clinical High Risk for Psychosis (CHR), also known as prodromal, At-Risk Mental State (ARMS), and Ultra High Risk (UHR), denotes an elevated risk of developing psychosis^1^. CHR individuals are assessed using the Comprehensive Assessment of At-Risk Mental States (CAARMS) assessment tool^2^ to determine one of three syndromes: attenuated positive symptoms (APS) (sub-threshold psychotic symptoms), brief limited intermittent psychotic symptoms (BLIPS) (brief psychosis lasting less than one week), or genetic risk and/or deterioration (GRD) (Positive family history of psychosis plus a decline in functioning)^2^. The CHR concept was devised in order to identify at-risk individuals thereby affording the opportunity for preventative strategies^3^.

Psychosis has been deemed to be a multifactorial polygenic disorder with heritability estimates ranging between 31% and 44%^4–6^. While this finding suggests that the etiology of psychosis involves a significant genetic contribution, environmental insults are also thought to play a critical role. Adverse life events have repeatedly been implicated in the development of First Episode Psychosis (FEP)^7^ with 89% of FEP patients reporting one or more adversities compared to 37% of controls^8^. Specifically, childhood/adolescent sexual, physical, and emotional abuse, physical/emotional neglect, separation, and institutionalization were 4–17 times higher for the FEP group. Moreover, for each additional adversity, the risk of psychosis increased 2.5 times^8^. Similarly, CHR individuals were found to have experienced significantly more severe adverse events than controls, regardless of trauma subtype^9^. Specifically, CHR individuals were 5.5, 2.5, and 3.1 times as likely to report emotional abuse, physical abuse, and bullying victimization, respectively^9^. Yet, it is clear that not everyone who develops psychosis has experienced severe adversity, such as abuse, neglect, or separation, and that other environmental factors may also play a significant role. Specifically, *psychosocial stress* is emerging as a possible contributory factor in the onset of psychotic-like experiences in CHR individuals^10^. Psychosocial stress has been defined as any social or cultural situation that causes physical, emotional, or psychological strain on an individual^11^. The physiological effects of psychosocial stress include increased heart rate and variability, skin conductance, decreased brain volumes, inflammation, alteration in hypothalamic–pituitary adrenal axis function, and increased cortisol secretion^12^. Psychological consequences of psychosocial stress include reduced self-esteem and motivation, increased negative affect, aggression, and withdrawal from social situations^12^. These negative effects can increase the risk of psychopathology, which is consistent with the Stress-Vulnerability Model^13^. This model posits that an individual’s predisposing bio-psychosocial vulnerability (biological, psychological, and social risk factors) interacts with stress caused by various life experiences leading to the manifest illness such as depression, anxiety, as well as psychosis^13^. Therefore, an individual with a high bio-psychosocial vulnerability will only need to experience a low level of (internal or external) stress in order to develop psychosis; while in contrast, an individual with a low overall level of bio-psychosocial vulnerability will need to experience a high level of stress in order to manifest the illness. Behavioral Sensitization has been proposed as a possible mechanism to account for the relationship between stress and psychosis symptoms^14^. This notion suggests that cumulative exposures to environmental insults produces an increased sensitivity to stress and elevated emotional responses to similar stressors subsequently experienced^14^. Indeed, early experiences of trauma and life events have been found to contribute to increased stress sensitivity in adulthood^15–17^ and patients with psychosis have been found to react with more intense emotions to perceived stress in daily life compared to controls^18,19^, giving credence to the behavioral sensitization concept. Therefore, the stress-vulnerability model denotes the effects of cumulative stress on a pre-existing trait vulnerability, while the concept of stress sensitivity refers to the magnitude of affective arousal in response to repeated stressors.

Exposure to psychosocial stress has been found to be higher in the CHR individuals compared to the general population^20^. Contrarily, another study found that the exposure to psychosocial stress may actually be comparable between these groups but was found to have a greater negative impact in the CHR group^21^. Indeed, a greater perception of psychosocial stress was associated with more severe positive symptoms in a CHR group compared to help-seeking controls^22^. Greater subjective distress in response to psychosocial stressors in CHR individuals may in part be accounted for by individual constitutional characteristics such as personality and temperament^22^, in addition to their level of sensitization, whereby repeated exposure to stress leads to an elevated affective response to subsequent stressors^14,23^. It is important to note that most measures of stress do not differentiate general stressors from psychosocial stressors perhaps because most, if not all, stressful events would contain a social component, highlighting the difficulty in ascertaining the differences between these two types of stressors. However, it is possible that they may exert similar effects on the stress response system if they are both appraised as challenging, threatening, or harmful^24^. It has therefore been suggested that the appraisal of an event as stressful, rather than the type of event, may be important in understanding the relationship between stress and the onset of psychosis^25^.

In regards to possible constitutional factors, interpersonal sensitivity refers to the undue and excessive awareness of, and sensitivity to, the behavior and feelings of others. This concept comprises of interpersonal awareness, a fragile inner self, need for approval, separation anxiety, and timidity^26^. High levels of interpersonal sensitivity have been characterized by avoidant behaviors such as social withdrawal and appeasement behaviors so as to avoid conflict or rejection by complying with the expectations of others^27^. The aforementioned coping strategies employed by individuals with high interpersonal sensitivity inadvertently affects social performance and functioning^28^.

Alongside personality traits, behaviors such as social withdrawal have also been investigated in psychosis. Social withdrawal can be defined as retreat from interpersonal relationships usually accompanied by an attitude of indifference and detachment^29^. Social withdrawal often leads to social isolation, loneliness, disturbed sleep hygiene, loss of support, and the development of psychiatric conditions^30^. Substance misuse, mood disorders, and psychotic disorders are one of many psychopathologies that can be associated with avoidant behaviors such as social withdrawal. In relation to psychosis, some have suggested that it precedes its onset^31^, while others argue it is a consequence of the disorder^32^. Compared to controls, CHR individuals exhibit greater levels of social withdrawal^33^, which is associated with increased symptomatology, such as positive and negative symptoms, reduced psychosocial and occupational functioning, and increased suicidal thoughts and substance misuse^30^. These behaviors in turn may contribute to the formation and persistence of psychosis symptomatology. Indeed, higher levels of social withdrawal have been associated with an increased likelihood of transition to psychosis^34^.

Psychosis symptomatology therefore appears to be influenced by a plethora of social factors such as psychosocial stress, interpersonal sensitivity, and social withdrawal. The aforementioned social factors have been observed to influence the development of symptoms and the progression of the illness, as well as impacting on long-term outcomes. Nevertheless, the reliability of these associations within the literature remains unclear. Furthermore, it remains unclear whether social factors are precipitating factors, perpetuating factors, or both. Therefore, a synthesis of the existing literature may help to elucidate the influence of psychosocial stress in individuals at CHR and its role in the transition to psychosis. To date, no systematic review has been conducted on the impact of different types of psychosocial stressors in CHR individuals incorporating the role of social behaviors and personality characteristics.

Therefore, the aim of this review was to summarize the existing evidence regarding the relationship between psychosocial stress, interpersonal sensitivity, and social withdrawal on transition to psychosis in CHR individuals. The following outcomes will be considered in the included papers: CHR status vs. controls, psychosis/affective symptomatology, and rate of transition to psychotic disorders.

## Method

### Protocol and registration

This review was conducted in accordance with the Preferred Reporting Items for Systematic Reviews and Meta-Analyses (PRISMA) guidelines^35^. Methods and inclusion criteria were specified in advance and documented in a protocol registered with the International Prospective Register of Systematic Reviews (PROSPERO; PROSPERO registration: CRD42021264478).

### Search strategy and selection criteria

A systematic search of Ovid (PsychINFO, EMBASE, MEDLINE, and GLOBAL HEALTH) was conducted, including studies from database conception to February 28, 2022. The following search strings were used: (at-risk mental state OR ultra-high risk OR clinical high risk OR attenuated psycho* OR prodrom* OR transition or conver* or psycho*) AND (interpersonal sensitiv* OR interpersonal awareness OR relational sensitiv* OR social withdrawal OR social avoidance OR social network OR social stress* OR social advers* OR psychosocial stress*).

**Fig. 1:**
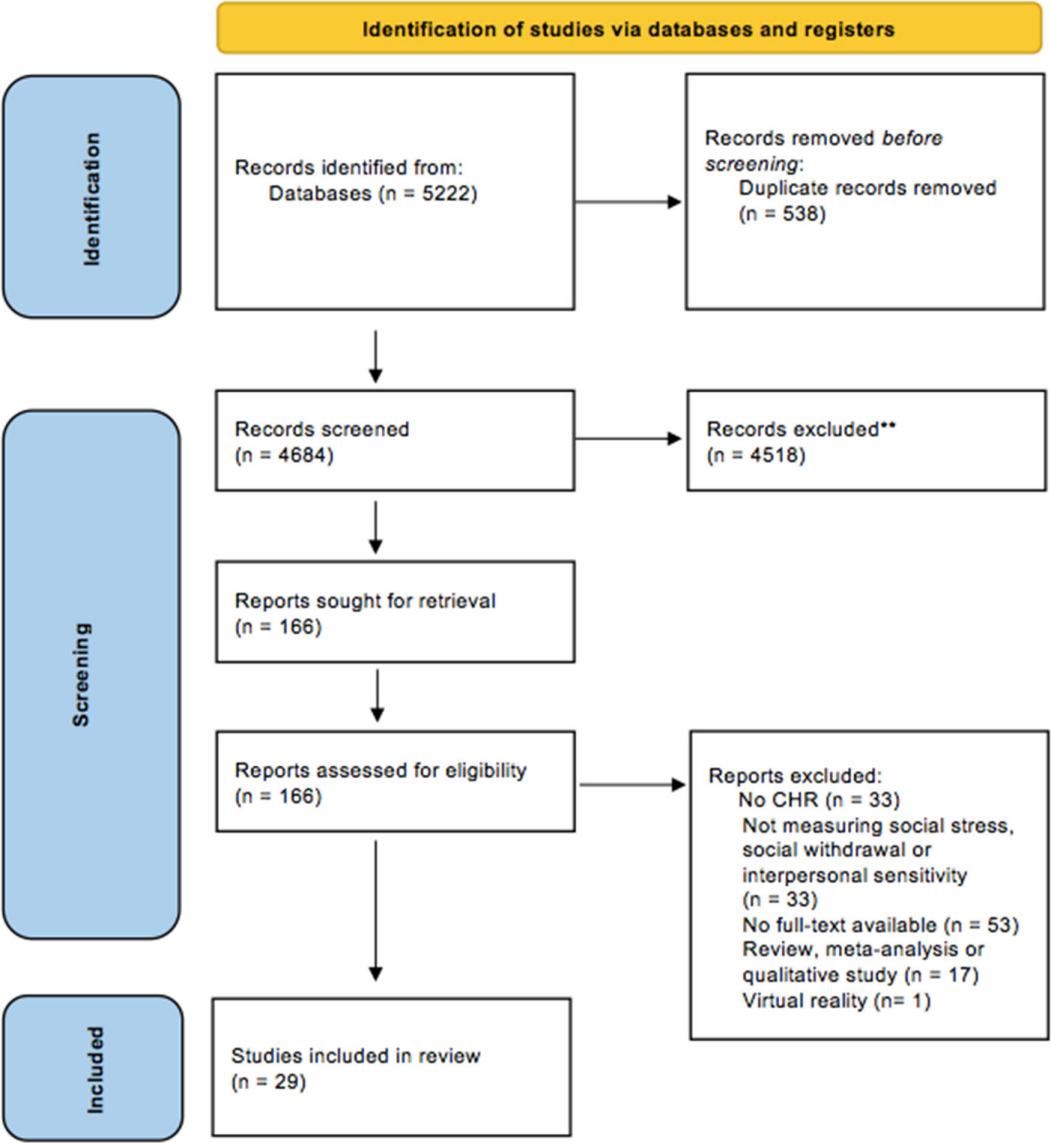
PRISMA flow diagram.

Any length of follow-up and any date of publication were included. Eligible studies measured psychosocial stress, interpersonal sensitivity, or social withdrawal and CHR status. Studies written in languages other than English and conference abstracts were excluded from the review. The study selection process is summarized in the PRISMA flow diagram (see Fig. 1).

### Data extraction process

A data extraction form was developed in Microsoft Excel. One review author (PR) extracted the following data from the included studies: (1) participant characteristics (CHR status) and the included paper’s inclusion and exclusion criteria; (2) outcome measures (psychosocial stress, interpersonal sensitivity, social withdrawal); (3) additional outcomes (including symptom severity, transition to psychosis, early/recent trauma); (4) statistical analysis used; (5) risk of bias assessment outcome; and (6) main findings (including means, standard deviations, effect sizes, and confidence intervals, where available). Study screening was performed independently by one reviewer and was subsequently cross-checked by a second reviewer (KM). Disagreements were resolved by consensus. All study designs were included in the review, except case studies.

The Grading of Recommendations Assessment, Development and Evaluation working group methodology^36^ was employed to assess the quality of evidence by examining the following domains: risk of bias, consistency, directness, precision, and publication bias. The quality of evidence was therefore rated as high, moderate, low, or very low. The risk of bias and certainty assessment was also performed.

### Analysis

Due to the expected low power and sparsity of literature, a descriptive summary of findings was planned. Summaries of each study were written in a Microsoft Word document, which, combined with the data extraction form and the Grading of Recommendations Assessment, were used to draw out analytical themes. No additional analyses were conducted.

## Results

A total of 5222 articles were retrieved during the initial search and 4684 articles remained following de-duplication. Through the initial screening involving title and abstract review 4518 articles were removed, thus 166 full-text evaluations took place. The full-text evaluations resulted in 29 articles matching the inclusion criteria for this review. Articles were excluded if they did not examine CHR status and at least one of the following variables: psychosocial stress, interpersonal sensitivity, or social withdrawal. Studies were also excluded if no full-text was available, if it was classified as a conference abstract, was not written in English, and if the study examined virtually constructed social situations rather than real-life occurrences.

### Study characteristics

This review included 29 studies published from 1999 to 2021, with most of the studies published from 2011 onwards. Out of 29 studies, 25 of them were carried out in Western countries (USA, Canada, UK, Netherlands, Italy, and Australia), while only 4 studies were carried out in non-Western countries (China, Seoul, and Brazil). The total sample size of included studies was *n* = 3143. The sample size ranged from *n* = 25–764, with the average sample size consisting of 108 participants. The participants were mainly recruited through Universities or specialized clinics and GP referrals, while all the assessors possessed a relevant postgraduate or doctoral degree. Out of all the included studies, 16 were cross-sectional in design, whereas 13 were longitudinal (1 to 5-year follow-up). In addition, one study employed a combined cross-sectional and longitudinal design.

### Population characteristics

Participants’ age ranged from 16 to 29 (average = 20). In terms of gender, males comprised more than 50% of the sample in all of the studies. The measurement of socio-economic status was not reported in 6 studies, while other studies measured educational achievement, employment status, or social class. The average years spent in education for CHR subjects was ~13.69, which was reported in 4 studies, while 6 studies looked at education level through the highest level of attainment. Three studies reported that the highest level of education was high school, while 3 studies reported it as university completion. Eight studies examined employment status of the clinical sample with 4 studies reporting higher rates of unemployment amongst CHR population and 4 studies reporting the opposite. The ethnicity of study participants was only reported in 13 studies. The samples were predominantly Caucasian, ranging from 50–80% of the sample. The exception was two studies^37^^,^^38^, which included a higher proportion of ethnic minority participants. Lastly, control groups were demographically matched to the clinical sample.

### Clinical and psychosocial stress measures

The following measures were employed to determine CHR status and transition to psychosis: Structured Interview for Prodromal Symptoms (SIPS)^39^; Comprehensive Assessment of At-Risk Mental States (CAARMS)^2^; Prodromal Questionnaire (PQ)^40^; Schizophrenia Proneness Inventory-Adult^41^; Present State Examination (PSE)^42^; Scale of Prodromal Symptoms (SOPS)^43^; Diagnostic Statistical Manual-Fourth Edition^44^; Diagnostic Interview for Psychosis^45^; and the Structured Clinical Interview for DSM Disorders (SCID)^46^.

Furthermore, the frequency and severity of psychosocial stress was measured using the following outcome measures: Childhood Trauma and Abuse scale^47^; Behavior Assessment for Children-Second Edition (BACS-2)^48^; The Schedler-Westen Assessment Procedure-2000^49^; The Daily Stress Inventory^50^; Childhood Trauma Questionnaire^51^; Coddington Life Events Record^52^; Life Events Scale^53^; Kiddie Schedule for Affective Disorders and Schizophrenia-Present and Lifetime version^54^; Schedule of Recent Experiences^55^; Trier Inventory for Chronic Stress (TICS)^56^; Psychiatric Epidemiology Research Interview Life Events Scale^57^; and Individual and Structural Exposure to Stress in Psychosis-risk-states Interview^58^.

Lastly, the concepts of social withdrawal and interpersonal sensitivity were measured using the following scales: Premorbid Adjustment Scale^59^; Social Interaction Scale^60^; Social Functioning Scale^61^; and the Interpersonal Sensitivity Measure^26^.

### Risk of bias and certainty assessment

The risk of bias assessment is summarized in Table 1 for cross-sectional studies and Table 2 for longitudinal studies. The certainty assessment was conducted using the GRADE criteria and deemed the effects and conclusions of this systematic review as moderate. The moderate score was given due to the high consistency, precision, and directness found in the included studies. The potential for risk of bias was due to lack of blinding and the possible effects of confounding variables, which prohibited granting a higher-ranking score.

**Table 1:** The NOS assessment—cross-sectional studies.

	Selection		Comparability			Outcomes		
Study	Representativeness of the sample	Sample size	Non-respondents	Ascertainment of the exposure	Confounding factors controlled	Assessment of outcome	Statistical test	Total quality scores
Addington et al.^116^	0	1	1	2	2	2	1	9
Bentley et al.^37^	0	1	1	2	2	2	1	9
Boldrini et al.^117^	1	1	1	2	2	2	1	10
Chudleigh et al.^104^	0	1	1	2	1	2	1	8
Hodges et al.^118^	0	1	1	1	1	1	1	6
Huang et al.^119^	0	1	1	2	2	2	1	9
Loewy et al.^120^	0	1	1	2	2	2	1	9
Magaud et al.^121^	0	0	1	2	2	2	1	8
Masillo et al.^122^	0	1	1	2	0	2	1	7
Masillo et al.^123^	0	1	1	2	0	2	1	7
Mushtaq et al.^124^	1	1	1	2	2	2	1	10
Pruessner et al.^10^	0	1	1	2	2	2	1	9
Shim et al.^125^	0	1	1	2	0	2	1	7
Vargas et al.^126^	0	1	1	2	2	2	1	7

**Table 2:** The NOS assessment—longitudinal studies.

	Selection				Comparability		Outcomes			
Study	Representativeness of exposed cohort	Selection of non-exposed cohort	Ascertainment of exposure	Demonstration that outcome of interest was not present at start of study	Adjust for the most important risk factors	Adjust for other risk factors	Assessment of outcome	Follow-up length	Loss to follow-up rate	Total quality score
Carol^127^	1	0	1	1	1	1	1	0	1	7
De Vos et al.^128^	0	1	1	1	1	1	1	0	1	7
De Vylder et al.^129^	1	1	1	1	1	1	1	0	1	8
Dragt et al.^65^	0	0	1	1	1	0	1	0	1	5
Freitas et al.^130^	0	1	1	1	1	1	1	0	1	7
Jang et al.^131^	0	1	1	1	1	1	1	0	1	7
Kline et al.^38^	1	1	1	1	1	1	1	0	1	8
Kraan et al.^132^	0	1	1	1	1	1	1	0	1	7
Kraan et al.^133^	0	1	1	1	1	1	1	0	1	7
Masillo et al.^134^	1	1	1	1	1	1	1	0	1	6
Mason et al.^135^	0	1	1	1	0	0	1	0	1	5
Stowkowy et al.^62^	0	1	1	1	1	1	1	0	1	7
Trotman et al.^21^	0	1	1	1	0	1	1	0	1	6

### Effects of psychosocial stress, social withdrawal, and interpersonal sensitivity on psychosis risk

From the included studies, 16, 8, and 5 studies examined the effect of psychosocial stress, social withdrawal, and interpersonal sensitivity on risk of psychosis in individuals at CHR, respectively (see Tables 3–8).

**Table 3:** Psychosocial stress: characteristics of studies meeting inclusion criteria (*n* = 16).

Author (date) location	Study design sample (number & diagnosis) (gender ratio: M/F) (CHR/controls)	Mean age (SD) ethnicity (CHR/controls)	Diagnosis and manual used & comorbidities (CHR/controls)	Outcome measures	Main findings and clinical implications
Addington et al.^116^	Cross-sectional 360 CHR 180 HC 210 M/150 F 100 M/80 F	18.98 (SD = 4.18) 19.54 (SD = 4.78) Caucasian 55%/58.9%	Structured Interview for Prodromal Symptoms (SIPS) No comorbidities reported	Calgary Depression Scale (CDS) Brief Core Schema Scale (BCSS) Social Interaction Anxiety Scale (SIAS) Social Anxiety Scale (SAS) Perceived Discrimination Scale (PDS) Alcohol and Drug Use Scale Global Assessment of Functioning Scale: Social and Role (GAF)	CHR participants experienced significantly more types of trauma (*z* = −8.68, *P* < 0.05) and bullying (*z* = −4.89, *P* < 0.05) compared to controls. CHR females reported significantly more trauma than CHR males. Those who had experienced past trauma and bullying were more likely to have increased levels of depression and anxiety and a poorer sense of self. These results offer preliminary support for an association between a history of trauma and later subthreshold symptoms.
Bentley et al.^37^	Cross-sectional 36 CHR 60 HC (Help-seeking) 10 M/26 F 23 M/37 F	15.17 (2.44) 15.69 (3.18) African American 55%/49% Caucasian 30%/34% Multiracial or other 15%/17%	Structured Interview for Prodromal Symptoms (SIPS) APS: 78% GRDS: 14% BIPS: 8% Mood disorder: 45%/55% Anxiety Disorder: 12%/11% PTSD: 15%/11% AD/HD: 15%/10%	Behavior Assessment System for Children, Second Edition (BASC-2)	For CHR participants, parent–child relationships had a significant negative effect on social stress (*b* = −0.73, *t*[92] = − 3.77, *P* < 0.001, f2 = 0.15); no significant relation was evident for the help-seeking control participants (*b* = −0.15, *t*[92] = −0.94, *P* = 0.35, f2 = 0.01). These findings suggest that a positive parent–child relationship may be a protective factor against social stress for those at risk for psychosis. Findings provide additional evidence to suggest that interventions that simultaneously target both social stress and parent–child relationships might be relevant for adolescents and young adults at clinical high risk for psychosis.
Carol^127^	Longitudinal 57 CHR 66 HC 34 M/23 F 30 M/36 F	19.04 (1.63) 18.42 (2.46) Hispanic 11/17 Asian 3/7 Black 1/2 Caucasian 40/38 Interracial 2/2	Structured Interview for Prodromal Symptoms (SIPS) Mood disorders: 30% Anxiety Disorders: 33% PTSD: 7% ADHD: 12% No comorbidities reported in HCs	Daily Stress Inventory (DSI)	CHR individuals reported significantly higher frequency of stressful events [*t*(117) = 3.01, *P* = 0.003] and significantly higher levels of overall distress [*t*(117) = 4.47, *P* < 0.001] compared to HCs. CHR group showed significantly more positive [*t*(63) = 17.62, *P* < 0.001] and negative [*t*(58.19) = 11.08, *P* < 0.001] symptoms compared to HCs. CHR group showed significantly elevated resting cortisol levels compared with matched HC adolescents when controlling for age, [*F*(1,100) = 2.99, *P* = 0.044].
De Vos et al.^128^	Longitudinal 12 month follow up 81 CHR 32 M/49 F	18 (3.3) Ethnicity not recorded.	Comprehensive Assessment of At-Risk Mental State (CAARMS) APS: 82.7% GRDS: 7.4% BIPS: 0% Major Depressive Disorder: 64.2% Anxiety Disorder: 42% Dysthymic Disorder: 3.7% Eating Disorder: 1.2% Substance Use Disorder: 16% Other diagnoses: 2.5% Schizotypal Personality Disorder: 1.2% Borderline Personality Disorder: 6.2%	Childhood Trauma Questionnaire (CTQ) Brief Psychiatric Rating Scale (BPRS) Scale for the Assessment of Negative Symptoms (SANS) Structured Clinical Interview for the Diagnostic and Statistical Manual of Mental Disorders-IV (DSM-IV) for Axis I & Axis II disorders (SCID-I, SCID-II) Social and Occupational Functioning Scale (SOFAS) Global Functioning Scale (GFS) Social & Role	The majority of CHR sample (82.7%) experienced at least one form of early trauma. No significant correlations emerged between the CTQ total score and baseline levels of APS severity, distress associated with APS and functional outcomes. However, there was a significant positive correlation (*r* = 0.23, *P* = 0.044) between the CTQ total score and the CAARMS suicidality and self-harm baseline score. The high prevalence of Childhood Trauma (CT) in CHR individuals and its association with suicidality and self-harm underlines the importance of inquiring about CT during clinical assessments.
Freitas et al.^130^	Longitudinal 87 CHR 115 HC 29 M/58 F 50 M/65 F	24.78 (4.11) 25.09 (4.31) Ethnicity not recorded.	The Prodromal Questionnaire (PQ) Structured Interview for Prodromal Syndromes (SIPS) Scale of Prodromal Symptoms (SOPS) No comorbidities reported in HCs	Childhood Trauma Questionnaire (CTQ)	Compared to HCs, CHR individuals scored significantly higher on: physical abuse (mean rank: controls = 93.81, CHR = 111.67; *P* = 0.027), sexual abuse (mean rank: controls = 94.67, CHR = 110.52; *P* = 0.007) and emotional abuse (mean rank: controls = 85.93, CHR = 122.09; *P* < 0.001). No differences were found for physical and emotional neglect. Childhood trauma such as emotional, physical, and sexual abuse, and physical and emotional neglect appear to be associated with CHR status. In CHR individuals, physical abuse correlated with perceptual abnormalities, and physical neglect correlated with disorganized speech/thought, whereas physical and emotional neglect negatively correlated with grandiosity symptoms.
Huang et al.^119^	Cross-sectional 56 FEP 83 CHR 61 HC 37 M/19 F 44 M/39 F 32 M/29 F	26.5 (8.5) 28.8 (8.4) 31.3 (7.9) 55 Han Chinese/ 82 Han Chinese/ 60 Han Chinese	Structured Interview for Prodromal Syndromes (SIPS) No comorbidities reported in HCs	Positive and negative syndrome scale (PANSS) Global Assessment Function (GAF) Montgomery–Asberg Depression Rating Scale (MADRS) Childhood Trauma Questionnaire-Short Form (CTQ-SF) Life Events Scale (LES) Perceived Social Support Scale (PSSS)	In terms of the CTQ Total, FEP and CHR groups scored significantly higher than HCs (*P* = 0.002 and *P* < 0.001, respectively). CHR individuals scored significantly higher on emotional neglect and physical neglect compared than HCs (*P* < 0.001 and *P* = 0.009, respectively). CHR group experienced a significantly more total life events compared with HCs (*P* = 0.004). FEP and CHR groups scored lower on PSSS Total than HCs (*P* = 0.03 and *P* = 0.01, respectively). In Family Support (FS) sub-domain, CHR possessed poorer family support than HCs (*P* = 0.03). In the CHR group, CTQ Total showed positive correlation with SIPS Total (*P* < 0.05), while LES Total showed a positive correlation with SIPS Positive (*P* < 0.05), SIPS Total (*P* < 0.01) and GAF (*P* < 0.05). CHR individuals had more childhood trauma, more recent life events and less social support than HCs. There was no significant difference on childhood trauma, life events and social support between CHR and FEP groups. Emotional and physical neglect were more frequently prevalent in CHR group than HCs.
Kline et al.^38^	Longitudinal 60 CHR 65 HC Total sample (*n* = 125) 48 M/77 F	15.3 (2.58) 16.46 (3.13) Total sample (*n* = 125) African American 46.4% Caucasian 34.4% Native American 1.6% Asian 0.8% Multiracial/other 14.4 %	Structured Interview for Prodromal Syndromes (SIPS) Mood Disorder: 61%/49.2% Anxiety Disorder: 50.8%/35.4%, PTSD: 31.7%/24.6%, ADHD: 45.8%/46.2%, Substance use disorder: 8.5%/7.7%, other disorder: 3.4% /18.5%	Kiddie Schedule for Affective Disorders and Schizophrenia, Present and Lifetime version (KSADS-PL)	The proportion of individuals reporting at least one event was higher in CHR (85%) relative to HCs (64.5%; *χ*^2^[1] = 6.75), *P* = 0.01). The CHR group endorsed significantly more lifetime trauma exposures than the HC group (mean exposures for CHR = 2.02, SD = 1.63; mean exposures for HC = 1.45, SD = 1.52; *t*[12] = −1.98, *P* = 0.05) For CHR individuals non-violent traumas were significantly associated with grandiose thinking (*P* < 0.05).
Kraan et al.^132^	Longitudinal 4 Year follow up 113 CHR 50 M/63 F	23.5 (5.4) Dutch 54% Minority 46%	Comprehensive Assessment of At-Risk Mental State (CAARMS)	Childhood Trauma Questionnaire-Short Form (CTQ-SF) Beck Depression Inventory-II (BDI-II) Social Interaction Anxiety Scale (SIAS) Social and Occupational Functioning Scale (SOFAS)	The percentage of CHR individuals reporting childhood adversity was as follows: emotional abuse (46.7%), physical abuse (20.9%), sexual abuse (24.8%), emotional neglect (66.7%) and physical neglect (41.9%). No significant association was found between childhood adversity subscales and the severity of positive symptoms at baseline and at 4-year follow-up. No significant association was found between total childhood adversity scores and any of the childhood adversity subscales (emotional abuse, physical abuse, sexual abuse, emotional and physical neglect) and transition to psychosis at 4-year follow-up.
Kraan et al.^133^	Longitudinal 24 month follow up 259 CHR 48 HC 139 M/120 F 26 M/22 F	22.7 (4.5) 23.98 (4.33) Ethnicity not reported	Comprehensive Assessment of At-Risk Mental State (CAARMS) APS: 78.7% GRDS: 8.4% BLIPS: 5.7% APS & Genetic risk: 7.2% No comorbidities reported in HCs	Structured Clinical Interview for the Diagnostic and Statistical Manual of Mental Disorders-IV (DSM-IV) for Axis I & Axis II disorders (SCID-I) Childhood Trauma Questionnaire (CTQ) Cannabis Experience Questionnaire (CEQ)	The number of CHR individuals that transitioned to psychosis was 31 (11.9%): 11 transitioned within the first 6 months, 13 at 12 months, and 7 at 24 months). 54% of the CHR individuals had experienced at least one form of childhood maltreatment compared to 17.4% of HCs (*P* < 0.001). This difference was apparent for each form of child maltreatment: emotional abuse; cases = 62.5%, controls = 27.1% (*P* < 0.001); emotional neglect; cases = 76.4%, controls = 33.3% (*P* < 0.001); physical abuse; cases = 24.3%, controls = 8.3% (*P* = 0.014); None of the univariate odds ratios for the association between each individual subtype of maltreatment and transition to psychosis was statistically significant. In addition, total child maltreatment did not increase the risk for transition to psychosis (OR = 2.46, 95% CI = 0.95 to 6.41, *P* = 0.065). Examination of the adjusted odds ratios showed that, after controlling for the other subtypes, a history of emotional abuse significantly contributed to transition (OR = 3.78, 95% CI = 1.17–12.39, *P* = 0.027)
Loewy et al.^120^	Cross-sectional 103 CHR 49 M/54 F	18 (4.2) Caucasian 52% Asian 18% Black/African-American 5% Native American/Other Pacific islander 1% Multiracial 16% Unknown or unreported 7%	Structured Interview for Prodromal Syndromes (SIPS)	Scale for Assessment of Psychosis Risk Symptoms (SOPS) Traumatic Events Screening Inventory for Children (TESI-C) Global Functioning: Role and Social Scales,	61% of CHR individuals reported lifetime exposure to traumatic events CHR individuals with a trauma history had significantly more severe perceptual disturbances and general/affective symptoms on the SOPS, as well as lower functioning ratings than CHRs without trauma. The number of traumatic events was significantly correlated with more severe perceptual disturbances, general/affective symptoms and lower functioning ratings. The number of interpersonal traumatic events was significantly correlated with more severe suspiciousness, perceptual abnormalities, general/affective symptoms and lower functioning scores.
Magaud et al.^121^	Cross-sectional 50 CHR 25 M/25 F	16.7 (3.3)	Structured Interview for Prodromal Syndromes (SIPS)	Childhood Trauma Questionnaire (CTQ) with added questions on cyberbullying	38% of CHR participants reported having experienced cyberbullying. The most frequent types reported were bullying via text messages, Facebook and instant messages (‘chat’). Bullying via texts and Facebook was associated with a past history of sexual abuse and physical neglect; Facebook with a past history of emotional neglect; and texts were more frequent in those who reported past physical abuse.
Pruessner et al.^10^	Cross-sectional 32 FEP 30 CHR 30 HC 16 M/14 F 15 M/15 F	20.33 (3.24) 22.47 (3.79) 80% Caucasian in both groups.	Comprehensive Assessment of At-Risk Mental State (CAARMS) *CHR comorbidities* Depression: 50% Dysthymia: 1% Anxiety: 37% Obsessive-compulsive disorder: 1% PTSD: 1% No comorbidities reported in HCs	Trier Inventory for the Assessment of Chronic Stress (TICS) Brief Psychiatric Rating Scale (BPRS) Global Assessment Function (GAF) Self-esteem Rating Scale (SERS) Brief COPE scale Multidimensional Scale of Perceived Social Support (MSPSS)	CHR reported significantly higher stress levels compared to FEP patients. Both patient groups showed lower self-esteem compared to controls, and the CHR group reported lower social support and active coping than controls. In the CHR group, higher stress levels and lower self-esteem were associated with more severe positive and depressive symptoms on the Brief Psychiatric Rating Scale. Multiple regression analyses revealed that stress was the only significant predictor for both symptom measures and that the relationship was not moderated by self-esteem. These findings show that CHR individuals experience high levels of psychosocial stress and marked deficits in protective factors. The results suggest that psychosocial interventions targeted at reducing stress levels and improving resilience in this population may be beneficial in improving outcomes.
Stowkowy et al.^62^	Longitudinal 2 year follow up 764 CHR 280 HC 436 M/328 F 141 M/139 F	18.50 (4.23) 19.73 (4.67) Caucasian 57.3%/54.3%	Structured Interview for Prodromal Syndromes (SIPS) No comorbidities reported in HCs	Childhood Trauma and Abuse scale	CHR individuals reported significantly more trauma, bullying and perceived discrimination than healthy controls. Only perceived discrimination was a predictor of later conversion to psychosis. Over the 2-year follow-up period, 86 participants transitioned to psychosis. More than half of CHR participants reported experiencing at least one type of bullying (53.3 versus 28.5 % for controls). Nearly half of the CHR participants reported experiencing at least one type of trauma (46.2 versus 11.4 % for controls), and more than half reported experiencing at least one type of discrimination (72.4 versus 57.5% for controls).
Trotman et al.^21^	Longitudinal 24 month follow up 314 CHR 162 HC 184 M/130 F 78 M/84 F	18.99 (4.18) 19.54 (4.77) Ethnicity not recorded.	Structured Interview for Prodromal Syndromes (SIPS) Scale of Prodromal Symptoms (SOPS) No comorbidities reported in HCs	Daily Stress Inventory (DSI) Life Events Scale (LES)	CHR individuals reported exposure to more Life Events (LE) compared to HCs. CHR individuals rated events as more stressful, and those who progressed to psychosis reported a greater frequency of LE and greater stress from events compared to those whose prodromal symptoms remitted. There was also some evidence of stress-sensitization; those who experienced more stress from LE rated current Daily Hassles (DH) as more stressful. The results indicate that the “prodromal” phase is a period of heightened stress and stress sensitivity, and elevated cumulative lifetime exposure to stressful events may increase reactions to current stressors.
Vargas et al.^126^	Cross-sectional 35 CHR 28 HC 17 M/18 F 6 M/22 F	20.63 (1.91) 20.04 (2.12) East Asian 8.6%/14.3% South Asian 5.7% Black 28.6%/14.3% Central/South American 8.6%/3.6% Caucasian 40%/39.3% Interracial 8.6%/10.7%	Structured Interview for Prodromal Syndromes (SIPS) No comorbidities reported in HCs	The Individual and Structural Exposure to Stress in Psychosis-risk states (ISESP) scale	Significant group differences were not observed for lifetime cumulative events, though CHR trended toward endorsing more events and greater stress severity. For stress severity across development, there were trending group differences for the 11–13 age range, and significant group differences for the 14–18 age range; notably, comparisons for earlier time points did not approach statistical significance. Associations between negative symptoms and cumulative severity of exposure were observed. These results suggest exploring exposure to cumulative environmental risk factors/stressors and stress severity across developmental periods and may inform predictive models and diathesis-stress psychosis-risk conceptualizations.
De Vylder et al.^129^	Cross-sectional and Longitudinal 4 year follow up 65 CHR 24 HC 50 M/15 F 14 M/10 F	19.5 (3.7) 20.4 (3.4) Caucasian 46.2%/66.7% African American 29.2%/20.8% Asian American 6.2%/4.2% More than one race 18.5%/8.3% Hispanic 33.9%/29.2%	Structured Interview for Prodromal Syndromes (SIPS) Scale of Prodromal Symptoms (SOPS) CHR individuals: 14% receiving antipsychotics 19% receiving antidepressants	Coddington’s Life Events Record The modified global assessment of function (GAF-m)	CHR individuals demonstrated impaired stress tolerance, which was associated over time with positive and negative symptoms, in addition to depression, anxiety, and poor function. By contrast, life events were comparable in CHR and HCs, and bore no association with symptoms. In this treated cohort, there was a trajectory of improvement in stress tolerance, symptoms and function over time. Impaired stress tolerance was associated with a wide range of “prodromal” symptoms, consistent with it being a core feature of the psychosis-risk state. Self-reported life events were not relevant as a correlate of clinical status. As in other treated CHR cohorts, most patients improved over time across symptom domains.

*APS* Attenuated Positive Syndrome, *BIPS* Brief intermittent psychotic symptoms syndrome, *BLIPS* brief limited intermitted psychotic symptoms, *CHR* Clinical High Risk, *FE*P First Episode Psychosis, *GRDS* Genetic Risk & Deterioration Syndrome.

**Table 4:** Social withdrawal: characteristics of studies meeting inclusion criteria (*n* = 8).

Author (date) location	Study design sample (number & diagnosis) (gender ratio: M/F) (CHR/controls)	Mean age (SD) ethnicity (CHR/controls)	Diagnosis and manual used & comorbidities (CHR/controls)	Outcome measures	Main findings & clinical implications
Boldrini et al.^117^	Cross-sectional 58 CHR 60 HC with PD 59 HC without PD 28 M/30 F 30 M/30 F 21 M/38 F	16 (1.6) 16 (1.6) 16 (1.4) Ethnicity not recorded.	Structured Interview for Prodromal Syndromes (SIPS) *CHR diagnoses* 14 GAD 10 Panic disorder, 6 Dysthymia, 6 Major depressive disorder *PD group diagnoses* 9 Cluster A 28 Cluster B 23 Cluster C *HC comorbidities* 14 GAD, 11 Eating disorder, 10 Panic disorder, Dysthymia, 6 Major depressive disorder, 6 ADHD 5 Oppositional defiant disorder	Shedler–Westen Assessment Procedure for Adolescents (SWAP-200-A)	CHR group had significantly higher mean scores in the SWAP- 200-A Schizoid and Schizotypal PD scales than the PD and HC without PD groups. The CHR and PD groups had significantly higher mean scores in the SWAP-200-A Borderline and Avoidant PD scales and lower mean scores in the SWAP-200-A High-Functioning scale than the HC without PD group. The SWAP-200-A items that had the highest mean scores and were most descriptive of personalities of the CHR group included: avoidance of interpersonal relationships, associated with feelings of shame, shyness, embarrassment, and fear of rejection, a tendency to express suspicion toward others, obsessional thoughts, severely impaired mentalization, in both self-oriented and other-oriented dimensions, emotional dysregulation, with dysphoric feelings of anxiety, and depression, odd and anomalous reasoning or perceptual experiences, especially when under stress; dissociative symptoms of depersonalization and derealization, and negative symptoms of avolition, abulia and blunted affect, and impaired role and academic/occupational functioning.
Chudleigh et al.^104^	Cross-sectional 20 FEP 20 CHR 20 HC 11 M/9 F 13 M/7 F 10 M/10 F	22.05 (3.0) 20.75 (2.7) 22.00 (2.5) Ethnicity not recorded.	Comprehensive Assessment of At-Risk Mental State (CAARMS)	Brief Psychiatric Rating Scale (BPRS) Social Functioning Scale (SFS) World Health Organization Disability Assessment Scale II (WHODAS) Social and Occupational Functioning Assessment Scale (SOFAS) Depression Anxiety Stress Scale (DASS) Brief Social Phobia Scale (BSPS)	CHR and FEP group’s SOFAS scores did not significantly differ from each other, but both groups were rated as functioning at a significantly lower level than HCs. HCs performed significantly better on interpersonal communication than the CHR group, as they were communicating more frequently with others. On the WHODAS, the CHR and FEP groups reported experiencing significantly more difficulty on the following subscales: understanding and communication, self-care, getting along with people, life activities, and participation in society, compared with HCs. The CHR and FEP groups did not differ from each other on any of these subscales. For the CHR group, increased levels of depressive symptoms were associated with decreased levels of both quantitative and qualitative measures of social functioning (i.e., withdrawal/social engagement, interpersonal communication and getting along with people). Significant correlations were found only between positive symptoms and the qualitative measures of disability; three of these four associations were for the CHR group only. Specifically, those at risk who reported more positive symptoms also indicated more difficulty with self-care (*r* = 0.68, *P* < 0.001), participating in life activities (*r* = 0.58, *P* < 0.01) and participation in society (*r* = 0.53, *P* < 0.01). There were no significant correlations observed between positive symptoms and any of quantitative social functioning (SFS) scores for CHR or FEP group.
Dragt et al.^65^	Longitudinal 36 month follow up 72 CHR 47 M/25 F	19.3 (4.0) 51 Caucasian 21 Other	Structured Interview for Prodromal Syndromes (SIPS) AS + BS: 48.6% AS: 22.2% BLIPS + AS + BS: 8.3% GRRF + AS + BS: 8.3% BLIPS + AS: 4.2% GRRF + AS: 4.2% BLIPS: 1.4% BLIPS + BS: 1.4% GRRF + BS: 1.4% Psychiatric medication: 41 Not on medication 17 Antipsychotic 7 Antidepressants 7 Other	Premorbid Adjustment Scale (PAS)	Over 36 months, 19 (26.4%) of the 72 included participants made the transition to psychosis. Social withdrawal significantly predicted transition to psychosis in CHR individuals (*P* = 0.001). Poor premorbid adjustment predicts onset of psychosis, as it may be a reflection of neurodevelopmental anomalies.
Hodges et al.^118^	Cross-sectional 100 CHR 32 HC 52 M/48 F 16 M/16 F	21.6 (2.8) 21.1 (2.2) Ethnicity not recorded.	Present State Examination (PSE) Schedule for Affective Disorders and Schizophrenia - Lifetime Version (SADS-L) *CHR Comorbidities* Major depressive syndrome: 1%, Minor depressive disorder: 4%, Alcohol dependency: 1%, obsessive-compulsive disorder: 1%, Suicidal behavior: 3%, GAD/Panic: 2% Antisocial personality disorder: 2% No comorbidities reported in HCs	Structured Inventory for Schizotypy (SIS)	Compared to HCs, CHR individuals demonstrated a trend towards significance for the following variables of the SIS: Childhood social isolation *P* = 0.08), Interpersonal sensitivity (*P* = 0.09), Social isolation (*P* = 0.09), Suicidal ideation (*P* = 0.09), Restricted affect (*P* = 0.08), Oddness (*P* = 0.05) and a significant association with Disordered speech (*P* = 0.04).
Jang et al.^131^	Longitudinal 12 month follow up 57 CHR 58 HC 37 M/20 F 40 M/18 F	21.3 (3.8) 20.8 (3.6) Ethnicity not recorded.	Comprehensive Assessment of At-Risk Mental State (CAARMS) Positive and Negative Syndrome Scale (PANSS) Psychiatric medication: 10 Antipsychotic (AP) 3 Antidepressants (AD) 2 AP + AD 2 Anxiolytics	Social Functioning Scale (SFS)	During the 12-month follow-up period, 13 of the 57 CHR individuals converted to full psychosis. The number of CHR individuals who converted to psychosis during the first 12 months was 6, between 12 and 24 months was 4, and after 24 months was 3. The mean time to conversion from inclusion in the study was 14.3 months. Both CHR groups, i.e., non-converters and converters, showed significantly lower average scores and significantly lower scores on the subscales addressing: social engagement/withdrawal, interpersonal behavior, independence—performance, independence—competence, and prosocial activities compared with HCs.
Mason et al.^135^	Longitudinal 12 month follow up 74 CHR 39 M/35 F	17.3 (2.8) Ethnicity not recorded.	PACE criteria APS: 58% GRDS: 26% BLIPS: 31% 20 No diagnosis, 16 Depression	Premorbid Social Adjustment scale (PSA) International Personality Disorder Examination (IPDE) Quality of Life Scale (QLS) Schedule of Recent Experience (SRE) Assessment of Prodromal and Schizotypal Symptoms (APSS) Brief Psychiatric Rating Scale (BPRS) Scales for Assessment of Positive and Negative symptoms (SAPS and SANS) Rating Scales for Depression & Anxiety (HRSD)/HRSA) Global Assessment of Functioning (GAF)	37 individuals (50%) made a transition to psychosis at 12-month follow-up The most reliable scale-based predictor was the degree of presence of schizotypal personality characteristics. Individual items assessing odd beliefs/magical thinking, marked impairment in role functioning, blunted or inappropriate affect, anhedonia/asociality and auditory hallucinations were also highly predictive of transition, yielding good sensitivity (84%) and specificity (86%) and odds ratio of 6.2. These predictors are consistent with a picture of poor premorbid functioning that further declines in the period up to transition.
Shim et al.^125^	Cross-sectional 32 CHR 30 HC 19 M/13 F 17 M/13 F	20.9 (3.9) 22.8 (2.4) Ethnicity not recorded.	Comprehensive Assessment of At-Risk Mental State (CAARMS) APS: 88% GRDS: 12% BIPS: 0% 13 CHR participants received antipsychotic medication.	Social Functioning Scale (SFS)	CHR group scored significantly lower than HCs on: Social engagement/withdrawal, Interpersonal behavior, and independence performance. Positive and negative symptoms were not significantly associated with social functioning, whereas disorganized and general symptoms were significantly correlated with poor “independence-competence” in CHR individuals.
Wisman van der Teen^64^	24 CHR 24 HC	21.78 (2.96) 20.27 (2.73)	Comprehensive Assessment of At-Risk Mental State (CAARMS) 16 CHR participants received medication 6 Atypical AP 1 Typical & Atypical AP, 6 SSRI, 3 Benzodiazepines	Experienced Sampling Method: Social context and frequency. Participants reported whether they were alone (i.e., “lone” and “alone with pet”) or they reported with whom they were (i.e., “classmates”, “friends”, “family”, “stranger”). The percentage of time spent alone was calculated and used as a measure of social withdrawal. Positive and Negative Syndrome Scale (PANSS) Green Paranoid Thoughts Scale (GPTS)	Social withdrawal did not significantly differ between CHR and HCs. CHR individuals showed no significant associations between social withdrawal and symptom severity nor with paranoia (PANSS: *b* = −0.11, *P* = 0.78; GPTS: *b* = −0.15, *P* = 0.47). An overall decrease of emotional distress in CHR individuals when with others was found, as indicated by a decrease in negative affect and an increase in positive affect when with others compared to when alone. Supporting patients to keep engaging in social interactions may alleviate their emotional distress.

*AP* antipsychotic medication, *APS* Attenuated Positive Syndrome, *AS* attenuated symptoms, *BS* basic symptoms, *BIPS* Brief intermittent psychotic symptoms syndrome, *BLIPS* brief limited intermitted psychotic symptoms, *CHR* Clinical High Risk, *GRDS* Genetic Risk & Deterioration Syndrome, *GRRF* genetic risk and reduced functioning, *SSRI* Selective Serotonin Reuptake Inhibitor.

**Table 5:** Interpersonal sensitivity: characteristics of studies meeting inclusion criteria (*n* = 5).

Author (date) location	Study design sample (number & diagnosis) (gender ratio: M/F) (CHR/controls)	Mean age (SD) ethnicity (CHR/controls)	Diagnosis and manual used & comorbidities (CHR/controls)	Outcome measures	Main findings and clinical implications
Hodges et al.^118^	Cross-sectional 100 CHR 32 HC 52 M/48 F 16 M/16 F	21.6 (2.8) 21.1 (2.2) Ethnicity not recorded.	Present State Examination (PSE) Schedule for Affective Disorders and Schizophrenia - Lifetime Version (SADS-L) *CHR Comorbidities* Major depressive syndrome: 1%, Minor depressive disorder: 4%, Alcohol dependency: 1%, obsessive-compulsive disorder: 1%, Suicidal behavior: 3%, GAD/Panic: 2% Antisocial personality disorder: 2% No comorbidities reported in HCs	Structured Inventory for Schizotypy (SIS)	Compared to HCs, CHR individuals demonstrated a trend towards significance for the following variables of the SIS: Childhood social isolation *P* = 0.08), Interpersonal sensitivity (*P* = 0.09), Social isolation (*P* = 0.09), Suicidal ideation (*P* = 0.09), Restricted affect (*P* = 0.08), Oddness (*P* = 0.05) and a significant association with Disordered speech (*P* = 0.04).
Masillo et al.^122^	Cross-sectional 62 CHR 39 HC 37 M/25 F 20 M/19 F	22.63 (4.05) 24.03 (4.22) Caucasian 51.6%/56.4% Asian 3.2%/12.8% Black 30.6%/23.1% Others 14.5%/7.7%	Prodromal Questionnaire (PQ)	Interpersonal Sensitivity Measure (IPSM) Ways of Coping Questionnaire (WCQ) Depression and Anxiety Stress Scale (DASS)	Compared to HCs, CHR individuals demonstrated higher scores on interpersonal sensitivity total score (*U* = 577.0, *P* < 0.001), interpersonal awareness (*U* = 592.0, *P* < 0.001), separation anxiety (*U* = 474.5, *P* < 0.001) and fragile inner self (*U* = 644.5, *P* < 0.001). Compared to HCs, CHR individuals demonstrated higher levels depression (*U* = 203.0, *P* < 0.001), anxiety (*U* = 241.0, *P* < 0.001) and stress (*U* = 335.5, *P* < 0.001) DASS subscales scores, and escape/avoidance WCQ (*U* = 537.0, *P* = 0.001). The higher the interpersonal awareness (rs=0.52, *P* = 0.001), separation anxiety (rs=0.71, *P* < 0.001), fragile inner self (rs=0.51, *P* < 0.001) and total IPSM (rs=0.52, *P* < 0.001) scores among CHR participants, the higher the level of paranoid ideas and suspiciousness. Higher sensitivity to interpersonal interactions, anxiety about separation from significant others and sense of having an inner or core self that is unlikeable and needs to be hidden from others were all associated with higher numbers of positive prodromal symptoms. Among participants with an CHR, total IPSM score (rs=0.40, *P* < 0.01), interpersonal awareness (rs=0.34, P < 0.01), separation anxiety (rs=0.50, *P* < 0.01) and fragile inner self (rs=0.35, *P* < 0.01) were significantly positively correlated with escape/avoidance WCQ subscale scores.
Masillo et al.^123^	Longitudinal 39 CHR 108 HC 21 M/18 F 48 M/60 F	17.36 (5.58) 18.51 (6.26) Ethnicity not recorded.	Prodromal Questionnaire (PQ) 20.5% prescribed psychiatric medication.	Interpersonal Sensitivity Measure (IPSM) Global Functioning: Social (GF: SS) Global Functioning: Role (GF: RS)	CHR individuals showed higher IPSM scores and lower GF: SS and GF:RS scores than HCs. A statistically significant negative correlation between two IPSM subscales (Interpersonal Awareness and Timidity) and GF: SS was found in both groups. Statistically significant correlations were found between interpersonal sensitivity and negative prodromal symptoms in both groups. In CHR individuals, the higher the sensitivity to interpersonal interactions (rs =0.292, *P* = 0.002), the anxiety about separation from significant others (rs =0.480, *P* = 0.002) and the sense of having an inner or core self that is unlikeable and needs to be hidden from others (rs =0.320, *P* = 0.047), the higher the level of negative prodromal symptoms. Interpersonal awareness and Timidity aspects of interpersonal sensitivity were associated with low levels of social functioning. Assessing and treating interpersonal sensitivity may be a promising therapeutic target to improve social functioning in CHR individuals.
Masillo et al.^134^	Longitudinal 18 month follow up 85 CHR 45 M/40 F	16.6 (5.05) Ethnicity not recorded.	Structured Interview for Prodromal Syndromes (SIPS) APS: 33.3% GRDS: 1.2% BLIPS: 0%	Interpersonal Sensitivity Measure (IPSM)	Interpersonal awareness (*P* ≤ 0.005) and separation anxiety (*P* ≤ 0.05) significantly correlated with SIPS general subscale at the 18-month follow-up, while fragile inner self showed a significant correlation with SIPS-positive subscale (*P* ≤ 0.05). Baseline need for approval and fragile inner self subscale’s scores explained 11% of the variance in the level of attenuated positive symptoms at follow-up (*F* (1, 81) = 2.753, *P* = 0.007, *R*^2^ = 0.115, *R*^2^ Adjusted =0.093). The analysis showed that fragile inner self was significantly associated with higher levels of attenuated positive symptoms at follow-up (*b* =0.306, *t*(2, 82) = 2.89, *P* = 0.005). Need for approval inversely influenced the level of attenuated positive symptoms at follow-up (*b* = −0.214, *t*(2, 82) = −2.01, *P* = 0.047). Baseline need for approval and interpersonal awareness subscale’s scores explained 16.3% of the variance in the level of general SIPS symptoms at follow-up (*F* (2, 81) = 7.95, *P* = 0.001, *R*^2^ = 0.163; adjusted *R*^2^ = 0.142.).
Mushtaq et al.^124^	Cross-sectional 25 CHR 25 HC Total sample 23 M/27 F	Total sample 24.38 (3.9) Ethnicity not recorded.	Schizophrenia proneness inventory-adult version (SPI-A). 26% Family history of psychiatric illness 74%. No family history of psychiatric illness	Interpersonal Sensitivity Measure (IPSM)	CHR demonstrated significantly higher levels of interpersonal sensitivity compared to HCs (*t* = −5.049, *P* < 0.00). Increased sensitivity to social interactions appears to be manifestation of the early phase of psychosis. Early intervention to those identified as sensitive to interpersonal relations may prevent transition to psychosis.

*APS* Attenuated Positive Syndrome, *AS* attenuated symptoms, *BLIPS* brief limited intermitted psychotic symptoms, *CHR* Clinical High Risk, *GRDS* Genetic Risk & Deterioration Syndrome.

**Table 6:** Findings of individual studies—psychosocial stress.

Study	Type of stressor	Higher levels of psychosocial stress compared to healthy controls	Associated with symptomatology (yes/no)	Associated with transition (yes/no)
Addington et al.^116^	Trauma	Yes	Yes	n/a
Bentley et al.^37^	Psychosocial stress	Yes	n/a	n/a
Carol^127^	Daily hassles	Yes	No	n/a
De Vos et al.^128^	Trauma	Yes	No	No
Freitas et al.^130^	Trauma	Yes	Yes	n/a
Huang et al.^119^	Trauma	Yes	n/a	n/a
Kline et al.^38^	Trauma	Yes	Yes	n/a
Kraan et al.^132^	Trauma	Yes	No	No
Kraan et al.^133^	Trauma	Yes	n/a	Yes
Loewy et al.^120^	Trauma	Yes	Yes	n/a
Magaud et al.^121^	Trauma	Yes	n/a	n/a
Pruessner et al.^10^	Psychosocial stress	Yes	Yes	n/a
Stowkowy et al.^62^	Trauma	Yes	n/a	Yes
Trotman et al.^21^	Daily hassles	No	n/a	Yes
Vargas et al.^126^	Significant life events	No	n/a	No
De Vylder et al.^129^	Significant life events	No	No	No

**Table 7:** Findings of individual studies—social withdrawal.

Study	Higher levels of social withdrawal compared to healthy controls	Associated with symptomatology (yes/no)	Associated with transition (yes/no)
Boldrini et al.^117^	Yes	n/a	n/a
Chudleigh et al.^104^	Yes	No	n/a
Dragt et al.^65^	Yes	Yes	Yes
Hogdes et al.^118^	Yes	n/a	n/a
Jang et al.^131^	Yes	n/a	n/a
Mason et al.^135^	Yes	n/a	Yes
Shim et al.^125^	Yes	No	n/a
Wiesman van der Teen et al.^64^	No	Yes	n/a

**Table 8:** Findings of individual studies—interpersonal sensitivity.

Study	Higher levels of social withdrawal compared to HC	Associated with symptomatology (yes/no)	Associated with transition (yes/no)
Hodges et al.^118^	Yes	n/a	n/a
Masillo et al.^122^	Yes	Yes	n/a
Masillo et al.^123^	Yes	Yes	n/a
Masillo et al.^134^	Yes	n/a	n/a
Mushtaq et al.^124^	Yes	n/a	n/a

### Psychosocial stress and psychosis risk

From the total of 16 studies, 13 studies reported higher levels of psychosocial stress in the CHR group compared to controls, while 3 studies found no difference between these groups (see Tables 3 and 6). The significant association was mainly driven by the presence of trauma (10 out of 13 studies), while the non-significant associations defined psychosocial stress as significant life events (2 studies) or as daily hassles (1 study).

All ten studies examining trauma noted significantly higher levels in CHR individuals compared to controls. Similarly, the two studies examining general psychosocial stress also demonstrated significant associations with CHR individuals compared to controls. However, the two studies investigating significant life events found no difference between CHR and control groups.

Nine studies measured the association between the psychosocial stress and symptom severity. Five studies found increased symptomatology (positive and/or negative) in CHR individuals who experienced greater rather than lower levels of psychosocial stress, and 4 studies found no relationship between psychosocial stress and symptom severity. Seven studies examined risk of transition, and 3 studies found an association between elevated psychosocial stress and increased risk of transition to psychosis^21,62,63^. Greater exposure to life events and distress associated with these events were found in CHR individuals who transitioned to psychosis compared to those that did not^21^, while emotional abuse increased the risk of transition to psychosis 3.8 fold in CHR individuals^63^. Perceived discrimination also increased risk of transition in CHR individuals by 52.4% for every unit increase in scores on lifetime perceived discrimination^62^.

### Social withdrawal and psychosis risk

Eight studies examined social withdrawal in CHR individuals (see Tables 4 and 7). All but one study^64^ reported higher levels of social withdrawal in individuals at CHR as compared to controls. One of the studies examined social withdrawal prior to CHR identification^65^ while the others measured its presence in individuals deemed to meet CHR criteria. Four studies examined the association between social withdrawal and psychosis symptomatology, two of which found a positive correlation, while the other two found no association between the levels of social withdrawal and the worsening of psychosis symptoms in CHR individuals. Two studies found higher levels of social withdrawal and subsequent conversion to psychosis.

### Interpersonal sensitivity and psychosis risk

Five studies examined interpersonal sensitivity in CHR individuals (see Tables 5 and 8). All five studies found significantly higher levels of interpersonal sensitivity in CHR individuals compared to controls. Only two studies examined interpersonal sensitivity in relation to psychosis symptomatology and a positive correlation was found for both positive and negative symptoms. Lastly, no study included in this review measured the effect of interpersonal sensitivity on transition risk.

### Bio-psychosocial model of transition to psychosis

The biological and neurodevelopmental vulnerabilities that play a key role in the stress-vulnerability model include hyperactivation of the hypothalamic–pituitary–adrenal (HPA) axis, dysregulation of neurotransmitters such as dopamine, GABA, and glutamate, aberrant salience, increased stress sensitivity and emotional reactivity to stressors, epigenetic effects (modification to the genome that affect gene expression without altering the DNA sequence), and gene-environment interactions (genetic factors influence the impact of an environmental exposure on an individual)^66–68^. Psychological factors include core beliefs and appraisals, emotions, attachment style, social cognition (set of neurocognitive processes related to understanding, recognizing, processing, and appropriately using social stimuli in one’s environment), and theory of mind deficits (the capacity to infer one’s own and other persons’ mental states)^69–73^; while social factors include the experience of trauma (physical, sexual, and emotional abuse, physical and emotional neglect, and parental loss), life events, and daily hassles^74–76^.

**Fig. 2:**
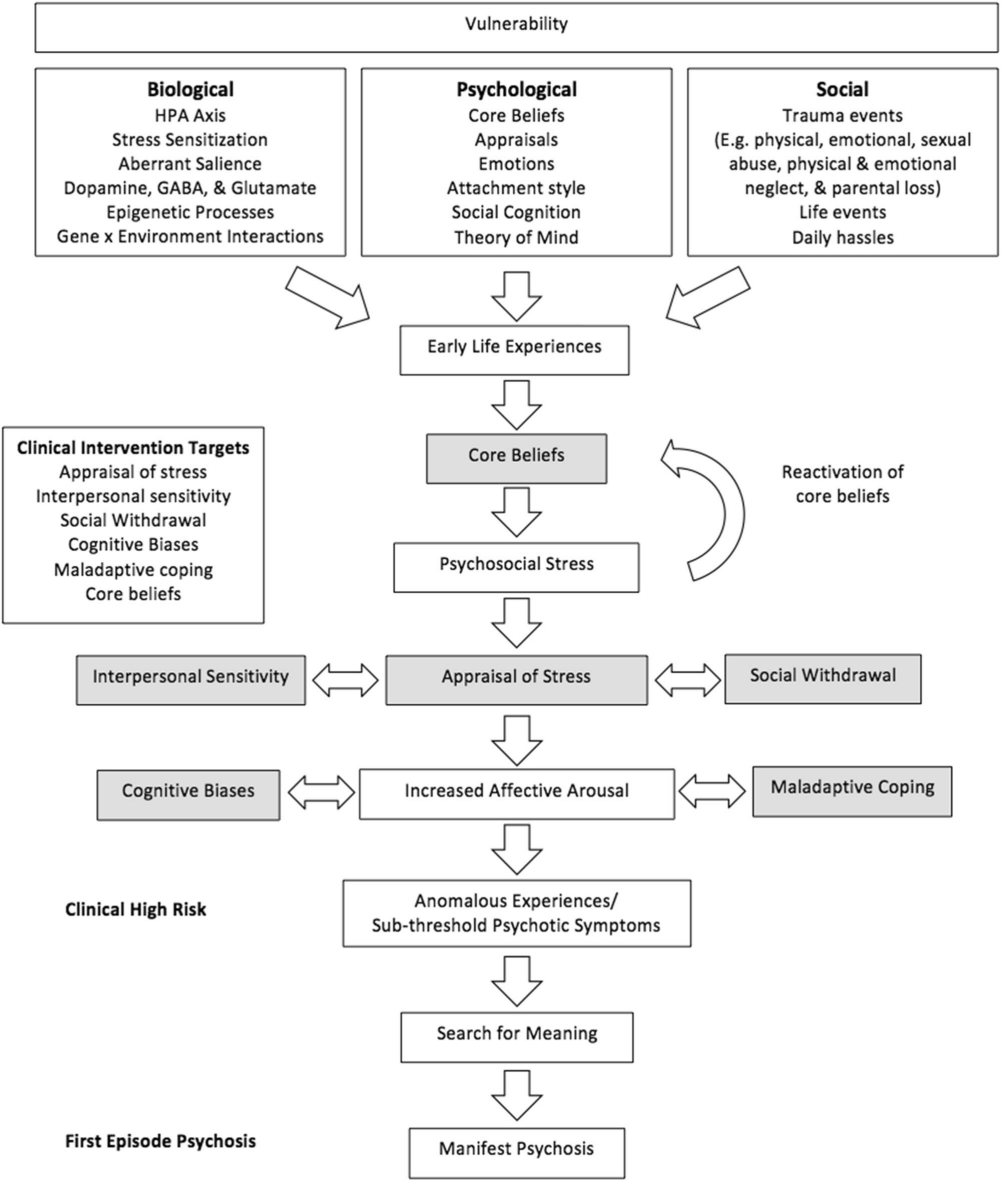
Bio-psychosocial model of transition to psychosis. A conceptual framework for integrating the influence of biological, psychological, and social factors in the transition to psychosis.

Based on the findings of the present paper, we propose the following *Bio-psychosocial Model of Transition to Psychosis* (see Fig. 2). This is a conceptual framework for integrating the influence of biological, psychological, and social factors in the transition to psychosis. Bio-psychosocial vulnerabilities, coupled with trauma/significant life events, lead to the formation of core beliefs, which are re-activated in response to psychosocial stressors. This core belief reactivation, in addition to the influence of interpersonal sensitivity, appraisals of stress, and social withdrawal, may give rise to increased affective arousal, which may be further amplified by the influence of cognitive biases and maladaptive coping. This affective pathway contributes to the generation of anomalous experiences/sub-threshold psychosis symptoms and the subsequent search for meaning for these unusual experiences, which may then lead to the formation of manifest psychosis. This model suggests that an underlying bio-psychosocial vulnerability coupled with exposure to trauma/significant life events during one’s early life leads to the formation of core beliefs about oneself, others, and the world (e.g., I’m worthless, others are untrustworthy, world is dangerous)^71^. Indeed, compared to healthy controls, CHR individuals reported significantly more negative beliefs about self and others and significantly less positive beliefs about self and others^77^. Moreover, negative core beliefs have been found to partially mediate the relationship between childhood trauma and persecutory beliefs and have been shown to be characteristic of patients with psychosis^78–81^. These authors concluded that these findings provide preliminary evidence about the cognitive mechanisms that may underlie the association between childhood trauma and later risk for psychosis.

Early experiences shape our attachment style and can lead to an interpersonally sensitive style of relating, which would make the individual hypervigilant to threat and hypersensitive to ambiguity and perceived rejection/harm from others^71,82,83^. Social withdrawal would remove opportunities to receive positive reinforcement/gathering corrective information, which would in turn foster ruminative/erroneous thinking, which would negatively impact one’s mood^84^. Psychosocial stress (trauma/significant life events/daily hassles) would drive subsequent appraisals of such events, such as “I am a failure/inferior/unlovable/vulnerable/weak” inducing dysregulation of the HPA axis and the dopaminergic system. Appraisals of stress may also influence social withdrawal and interpersonal sensitivity, which both may in turn further reinforce the appraisal of stress. For example, an interpersonal sensitive style of relating may misinterpret the actions of others as threatening/rejecting while ensuing social avoidance would preclude the receipt of disconfirmatory evidence, thereby maintaining the appraisal of stress.

In addition, if the psychosocial stress experienced later in life is thematically similar to the trauma/significant life events during one’s early life, it may serve to reactivate pre-existing core beliefs, further inducing a HPA stress response, elevated affective reactivity, and heightened stress sensitivity. Increased affective arousal/emotional dysregulation, in addition to the influence of cognitive biases, and maladaptive coping (e.g., rumination, substance misuse, emotion-oriented coping) may subsequently contribute to the formation of anomalous experiences as typified in the CHR state. One’s attempt to make sense of their experiences, otherwise known as their search for meaning, may then lead to the formation of manifest psychosis, such as delusions and hallucinations^85^. The search for meaning refers to the search for an explanation for anomalous experiences, recent events, or arousal and in doing so; pre-existing beliefs about self, world, and others are drawn upon^85^. These positive symptoms of psychosis in turn may reflect exaggerated themes of pre-existing insecurities and/or interpersonal concerns (e.g., repeated historical experiences of childhood physical abuse is likely to lead to the development of a vulnerability core belief, which would be reactivated by a critical incident concerning physical threat from others such as a mugging). This may give rise to persecutory delusions of threat of harm from others and congruent auditory hallucinations (e.g., voice saying “you’re in danger”, “don’t go outside”). Therefore, comprehensive assessments exploring the client’s early life experiences/significant life events, core beliefs, psychosocial stressors and associated theme (e.g., intrusion, harm, loss of control, helplessness, unlovability, worthlessness, weakness, inferiority), appraisals of stress, interpersonal sensitivity, social withdrawal, cognitive biases, maladaptive coping, affective responses, and emerging anomalous experiences/psychosis presentation (delusional and/or hallucinatory subtype) would aid in the development of personalised formulations and targeted interventions.

## Discussion

This systematic review sought to synthesize the literature examining the relationship between psychosocial stress and onset of psychosis in CHR individuals. Psychosocial stress, interpersonal sensitivity, and social withdrawal were higher in CHR individuals compared to healthy controls and there was some evidence of their association with positive symptoms of psychosis. There was also some evidence to support the role of psychosocial stress and social withdrawal in the transition to psychosis and no studies to date have examined the role of interpersonal sensitivity on transition to psychosis in CHR, which proposes avenues for future research.

While the present review focuses on the existing evidence of the relationship between psychosocial stress and risk of psychosis in individuals at Clinical High Risk (CHR) for psychosis, our companion review (Almuqrin et al. submitted) considers such evidence in individuals with a First Episode of Psychosis (FEP).

### Psychosocial stress

A repeated finding of the present review was for the association between higher levels of psychosocial stress in CHR individuals compared to controls. It is important to note that most studies driving this association examined trauma rather than significant life events or daily hassles, each of which are conceptually distinct constructs that need to be differentially defined and studied in relation to CHR symptomatology and conversion to psychosis. Psychosocial stress has been associated with increased positive and negative symptoms in individuals at CHR and appears to occur in a dose-response manner, as the greater the level of psychosocial stress experienced, the greater its impact on symptom severity^22^. This is in line with the dysregulation of the HPA Axis and the stress-vulnerability model regarding psychosis symptomatology arising from the cumulative effects of stress on a pre-existing bio-psychosocial vulnerability^13^. Interestingly, a lab-based study examining the effects of an experimentally induced psychosocial stressor found that CHR individuals produced higher overall cortisol levels from the pre-anticipation period through to the recovery period of the Trier Social Stress Test and exhibited higher levels of subjective stress prior to the stressor compared to controls^86^. These findings provide further support for the stress-vulnerability model and highlight the importance of examining the biological and subjective impact of psychosocial stress in CHR to further elucidate possible mechanisms of transition to psychosis. Environmental risks have also been found to act additively and synergistically with childhood trauma^87–89^ and stressful life events^90,91^, contributing to the persistence of sub-threshold psychosis symptoms in the general population. Psychosocial stressors such as childhood trauma and stressful life events have further been associated with higher levels of positive, negative, and depressive symptoms in the general population^92,93^. In addition, these psychosocial stressors, along with polygenic risk scores were found to exhibit independent additive effects on these three dimensions of subclinical psychosis^92,93^ further supporting the stress-vulnerability hypothesis. However, it is important to note that no gene-environment interaction was found^93^. These general population studies appear to parallel the CHR findings^22^ and are consistent with the stress-vulnerability and stress sensitivity models, whereby the cumulative effects of stress increase the likelihood of psychosis expression and increased affective arousal in response to such stressors, respectively. However, the finding of a positive correlation between psychosocial stress and positive psychosis symptoms in both CHR and non-CHR help-seeking controls^22^ suggests that the effects of psychosocial stress on psychosis symptomatology appears to be independent of clinical vulnerability to psychosis, thereby only providing partial support for the stress-vulnerability model. A bi-directional relationship may also occur in that psychosis symptomatology may also elicit unwanted interpersonal responses leading to further experiences of psychosocial stress^22^. It has also been found that compared to healthy controls, individuals at CHR are significantly more distressed by stressful events and that the appraisal of these events differentiated CHR individuals from controls^21,25^. However, it is important to note that to date, the majority of case-control studies examining CHR have utilized healthy non-clinical controls, as opposed to help-seeking (i.e., psychiatric) controls, which risks attributing group differences to psychosis-risk status rather than to non-specific psychopathology and comorbidities occurring in the CHR group^94^. The inclusion of both healthy controls and help-seeking samples in CHR would aid in elucidating a psychosis specific vulnerability, as opposed to a more general mental illness vulnerability^94^.

### Psychosocial stress and psychosis onset

Only three out of seven studies found a significant association between elevated psychosocial stress and increased risk of transition to psychosis^21,62,63^. Greater exposure to psychosocial stress, emotional abuse, and perceived discrimination were found to significantly increase the risk of transition to psychosis in CHR individuals compared to controls. It is important to note that the three studies that demonstrated significant associations with transition to psychosis had a 24-month follow-up period and sample sizes ranging from 259 to 764 CHR individuals and 162 to 280 controls, which contrasts the 35–105 CHR individuals and 24–28 controls in the non-significant studies and a shorter 12-month follow-up period. Therefore, the lack of significant findings regarding psychosocial stress and transition to psychosis in CHR may be due to existing studies being statistically underpowered to detect such effects and not having a long enough follow-up period to capture cases of transition.

### Interpersonal sensitivity

All five studies found significantly higher levels of interpersonal sensitivity in CHR individuals compared to controls. Higher levels of interpersonal sensitivity exhibited by CHR individuals compared to controls is congruent with the theory that certain personality characteristics may predispose individuals to mental illness, such as psychosis^95^. Indeed, interpersonal sensitivity has been associated with a greater severity of psychosis symptomatology. This finding implicates the important role of social interactions as a factor in influencing one’s well-being. Moreover, this relationship may indeed be bi-directional as individuals interact with their environment, which in turn impacts on the individual^66^. High interpersonal sensitivity can give rise to rumination about social performance and speech, preoccupation with the emotions displayed, and excessive focus on other people’s opinions, which may hinder social performance. Feelings of exclusion and perceptions concerning a lack of understanding from others can negatively impact on self-esteem, confidence, and motivation, which could exacerbate negative symptoms such as negative self-image, asociality, and avolition^96^.

In respect to positive symptoms of psychosis, increased levels of distress and subsequent avoidance of social situations could possibly account for their association with interpersonal sensitivity. The increased levels of distress could be attributed to one’s preoccupation with receiving negative social feedback and perceptions of a lack of approval from others. Preoccupation with social feedback would elicit both physiological and psychological reactions, thereby increasing levels of stress^97^. In regard to coping with stress, it has been found that CHR individuals reported feeling significantly more distressed by events, felt that they coped more poorly, and employed more emotion-oriented coping as opposed to task-focused coping^25^. These authors also found that compared to controls, CHR individuals were less likely to employ social diversion as a means of coping, which involves engaging with others to divert attention from stressors.

Even though the evidence from this review supports the concept of higher levels of interpersonal sensitivity leading to worsening symptomatology in CHR, it is useful to note that only two studies examined this association and further investigations are needed to examine the strength of this association.

No study to date has examined the relationship between interpersonal sensitivity and psychosis transition. The findings of increased severity of psychosis symptomatology in relation to interpersonal sensitivity might suggest the possibility of a direct link with conversion to psychosis. Nevertheless, it could also be possible that symptoms are affected by this personality trait but it may not extend to conversion to psychosis, as was the case for social withdrawal. Henceforth, without a number of studies investigating this aforementioned relationship, no conclusion can be drawn at this time.

### Social withdrawal

Compared to controls, social withdrawal was frequently observed in CHR individuals, who might avoid social situations due to emerging suspiciousness^98^. Moreover, auditory and/or visual hallucinations may directly impact the individual’s capacity to engage and follow a conversation, which may be distressing and may negatively impact self-esteem. Individuals may thus choose to avoid social situations to circumvent anticipated embarrassment and rejection from others^98^. Social avoidance/withdrawal may ensue failed attempts at social engagement or may be present pre-morbidly^99^. Unfavorable feedback from social interactions can also lead to social avoidance so as to avoid unpleasant feelings^100^. Social withdrawal results in a lack of social support, which in turn minimizes potential sources of external support that could challenge delusions and hallucinations, thus indirectly causing a greater reality mismatch^101^.

The mixed findings regarding the association between social withdrawal and increased symptomatology might be accounted for by the inclusion of varied outcome measures for social functioning. Interestingly, the two negative findings employed the Social Functioning Scale (SFS)^61^, which was validated on an outpatient schizophrenia sample with a mean illness duration of 8.8 years and assesses seven areas including social engagement/withdrawal, interpersonal behavior, prosocial activities, recreation, independence-competence, independence–performance, and employment/occupation. Of the two studies that found a positive association between social withdrawal and symptoms, one employed the Premorbid Adjustment Scale (PAS)^59^, which measures the level of functioning in four major areas: social accessibility-isolation, peer relationships, ability to function outside the nuclear family, and capacity to form intimate socio-sexual ties. The PAS may therefore be more nuanced and applicable to the CHR population compared to the SFS, possibly accounting for the significant positive finding. The other significant finding employed the Experience Sampling Method (ESM)^102^, which captures daily life data regarding social context and frequency, as well as emotional reactivity throughout the day for seven consecutive days. This is advantageous to questionnaire measures, as it allows for the investigation of experiences and interactions within a real-world context^103^. The mixed findings regarding the association between social withdrawal and increased symptoms of psychosis might also be accounted for by the included studies tending to examine positive rather than negative symptoms, highlighting the presence of a possible publication bias. It has been suggested that social withdrawal is more closely related to the latter rather than the former. Indeed significant associations have been found for social withdrawal and negative symptoms in CHR individuals^104^. The closer association between social withdrawal and negative symptoms may be due to the similarities in its characteristics. Social withdrawal is presumed to result from the lack of motivation to engage in social interactions, asociality, which is one of the primary negative symptoms^105^. Henceforth, the results might have been different if the association between social withdrawal and negative symptoms was assessed in the included studies.

The perception of social support may benefit reality testing, as it can weaken the intensity of delusions and hallucinations, subsequently improving insight in CHR^101^. If delusions and hallucinations remain unchallenged, they may intensify and exacerbate distress. It has also been posited that the lack of sensory stimulation derived from social interactions may contribute to an increase in hallucinatory experiences owing to the over-compensatory mechanisms of the nervous system^106^. In addition, socially withdrawn CHR individuals may not recognize their emerging illness and need for treatment, which would adversely affect their recovery^107^.

Two studies found higher levels of social withdrawal and subsequent transition to psychosis. The higher transition rates in CHR individuals who exhibit higher levels of social withdrawal can possibly be explained by a social network approach. Having social support can greatly impact one’s well-being in number of positive ways such as increasing self-esteem, improving confidence, providing an opportunity for new experiences, easing stress, and preventing loneliness. Alternatively, the lack of social networks can lead to social isolation, poor psychosocial functioning, and an increase in negative thoughts and feelings^108^. In addition, it has been suggested that higher transition rates are linked to a longer duration of untreated psychosis, as a result of a lack of social support. Having a close friend or supportive family environment can facilitate the more timely identification of behavioral change and mental health deterioration, which could then precipitate prompt engagement with services and the initiation of treatment^109^.

### Strengths and limitations

This is the first review to synthesize the literature regarding psychosocial stress, social withdrawal, and interpersonal sensitivity in CHR and highlights the importance of social factors in CHR symptomatology and their possible involvement in conversion to psychosis. However, this review has some limitations. The majority of studies included in the present review had a higher number of male than female participants. Poorer premorbid and psychosocial functioning, and increased substance misuse has been found in males compared to females, which would negatively impact on their ability to form social connections and to seek help from others^110^. Substance misuse may also negatively impact symptom severity, functioning, engagement with services, and recovery^111,112^. The included sample was predominantly Caucasian, which limits the representativeness and generalizability of the sample to Black and Minority groups, which have a higher incidence of psychosis^113^. Alongside the characteristics of the sample, the size of it could also have an impact on the results. Even though the average sample size was 100, 9 out of 27 studies had sample sizes under 100 participants. Small sample sizes can affect the reliability and representativeness of results. Additionally, the majority of the studies included in this review originated from Western countries, while a small number of studies were conducted in developing countries. This therefore limits the generalizability of the present findings^114^.

Furthermore, the present review examined three different types of psychosocial stress (trauma, significant life events, and daily hassles), however some studies did not specify the social stressor but referred to it by the collective name of social/psychosocial stress. Using this umbrella term limits the possibility of examining which factors influence CHR status and transition outcomes. Trauma, significant life events, and daily hassles represent conceptually distinct constructs that need to be differentially defined and studied in relation to CHR symptomatology and their role in transition. Therefore, specifying the type of stress would enhance the specificity of future investigations. In addition, it would be important to examine the effect of more recent and historical significant life events, as well as the appraisal of those events, as they may exert a differential influence on symptom formation and expression. Indeed, appraisals of stress have been found to differ between CHR individuals and controls and may impact on psychosis transition^25^.

### Clinical implications and future directions

This review synthesized the literature examining psychosocial stress, social withdrawal, and interpersonal sensitivity in individuals at CHR for psychosis. The negative impact of psychosocial stress on CHR individuals in terms of psychosis symptomatology and transition to psychosis emphasizes the importance of social factors in the CHR state. The proposed *Bio-Psychosocial Model of Transition to Psychosis* offers an explanatory framework for devising personalized, idiosyncratic, and symptom-specific formulations accounting for psychosis emergence in CHR and FEP. It highlights the possible mediating role of core beliefs in explaining the relationship between trauma and psychosis symptomatology, which would benefit from further investigation of core beliefs in CHR, FEP, and controls and their association with hallucinatory and delusion subtypes (e.g. persecutory, grandiose, religious, somatic, bizarre). This would subsequently inform psychosocial treatments, allowing for targeted interventions. For example, psychosocial treatments, such as Cognitive Behavior Therapy for Psychosis (CBTp) could employ this *Bio-Psychosocial Model of Transition to Psychosis* within collaborative case formulations with clients and could draw upon social aspects of treatment, such as social skills training, encouraging help-seeking behaviors, utilizing social support, and enhancing communication skills. This would also help to avoid the negative impact of social withdrawal. Additionally, Group CBTp could be employed to target social isolation and withdrawal, while also enhancing social skills in a group setting. Current interventions advocated for CHR include CBTp for alleviating symptoms and preventing transition to psychosis, as well as Cognitive Behavioral Family Interventions for Psychosis (CBFIP) in order to reduce stress, improve problem-solving, and enhance interpersonal communication skills^115^. However, neither intervention explicitly targets the appraisal of psychosocial stress and associated coping, which could improve social functioning in CHR individuals. This therefore affords an opportunity to devise and develop targeted CBTp interventions with an explicit focus on minimizing the effects psychosocial stress and improving social functioning. Additionally, as psychosocial stress is often unavoidable, learning to identify and challenge unhelpful appraisals and maladaptive coping leading to increased distress may enhance well-being. To date, there has been a lack of emphasis on the appraisals of stressful events, therefore their exploration in CHR, FEP, and controls in future studies are warranted. Interpersonal sensitivity could also be a therapeutic target, with the aim of elucidating vicious cycles and enhancing more adaptive behavioral coping methods of social integration rather than social avoidance. Therefore, identifying potential cognitive and affective mediators accounting for the relationship between psychosocial stress and psychosis could hold significant implications for the identification, prevention, and treatment of CHR individuals.

## Data Availability

Data sharing is not applicable to this article as no new data were created or analyzed in this study.
